# *In Silico* Computational Studies of Bioactive Secondary Metabolites from *Wedelia trilobata* against Anti-Apoptotic B-Cell Lymphoma-2 (Bcl-2) Protein Associated with Cancer Cell Survival and Resistance

**DOI:** 10.3390/molecules28041588

**Published:** 2023-02-07

**Authors:** Hittanahallikoppal Gajendramurthy Gowtham, Faiyaz Ahmed, Satish Anandan, C. S. Shivakumara, Ashween Bilagi, Sushma Pradeep, Chandan Shivamallu, Ali A. Shati, Mohammad Y. Alfaifi, Serag Eldin I. Elbehairi, Raghu Ram Achar, Ekaterina Silina, Victor Stupin, Mahadevamurthy Murali, Shiva Prasad Kollur

**Affiliations:** 1 Department of PG Studies in Biotechnology, Nrupathunga University, Nrupathunga Road, Bangalore 560001, India; 2 Department of Clinical Nutrition, College of Applied Health Sciences in Ar Rass, Qassim University, Al Qassim Region, Ar Rass 51921, Saudi Arabia; 3 Department of Clinical Nutrition and Dietetics, Sri Devaraj Urs Academy of Higher Education and Research, Kolar 563103, Karnataka, India; 4 Department of Integrative Medicine, Sri Devaraj Urs Academy of Higher Education and Research, Kolar 563103, Karnataka, India; 5 Department of Biotechnology and Bioinformatics, JSS Academy of Higher Education and Research, Mysuru 570015, India; 6 Biology Department, Faculty of Science, King Khalid University, Abha 9004, Saudi Arabia; 7 Cell Culture Lab, Egyptian Organization for Biological Products and Vaccines (VACSERA Holding Company), 51 Wezaret El-Zeraa St., Giza 12511, Egypt; 8 Division of Biochemistry, School of Life Sciences, JSS Academy of Higher Education and Research, Mysuru 570015, India; 9 Institute of Biodesign and Modeling of Complex Systems, I.M. Sechenov First Moscow State Medical University (Sechenov University), 119435 Moscow, Russia; 10 Department of Hospital Surgery, N.I. Pirogov Russian National Research Medical University, 117997 Moscow, Russia; 11 Department of Studies in Botany, University of Mysore, Manasagangotri, Mysuru 570006, Karnataka, India; 12 School of Physical Sciences, Amrita Vishwa Vidyapeetham, Mysuru Campus, Mysuru 570026, Karnataka, India

**Keywords:** B-cell lymphoma-2, binding affinity, *in silico*, molecular docking, *Wedelia trilobata*

## Abstract

In the present study, the binding affinity of 52 bioactive secondary metabolites from *Wedelia trilobata* towards the anti-apoptotic B-cell lymphoma-2 (Bcl-2) protein (PDB: 2W3L) structure was identified by using in silico molecular docking and molecular dynamics simulation. The molecular docking results demonstrated that the binding energies of docked compounds with Bcl-2 protein ranged from −5.3 kcal/mol to −10.1 kcal/mol. However, the lowest binding energy (−10.1 kcal/mol) was offered by Friedelin against Bcl-2 protein when compared to other metabolites and the standard drug Obatoclax (−8.4 kcal/mol). The molecular dynamics simulations revealed that the Friedelin-Bcl-2 protein complex was found to be stable throughout the simulation period of 100 ns. Overall, the predicted Absorption, Distribution, Metabolism, Excretion, and Toxicity (ADMET) properties of Friedelin are relatively better than Obatoclax, with the most noticeable differences in many parameters where Friedelin has no AMES toxicity, hepatotoxicity, and skin sensitization. The ADMET profiling of selected compounds supported their in silico drug-likeness properties. Based on the computational analyses, the present study concluded that Friedelin of *W. trilobata* was found to be the potential inhibitor of the Bcl-2 protein, which merits attention for further in vitro and in vivo studies before clinical trials.

## 1. Introduction

The B cell lymphoma 2 (Bcl-2) family proteins that appropriately regulate apoptotic cell death are associated with the progression and development of several cancers [1,2]. The first founding member of the Bcl-2 family of apoptosis regulators was the Bcl-2 protein, which protects the cells from apoptosis by directly binding to the pro-apoptotic Bcl-2 family members, thereby playing a crucial role in cancer cell survival and resistance in response to chemotherapy or radiation therapy [3]. Most prescribed chemotherapy drugs are synthetically prepared and have demonstrated their cytotoxic effects, not only on cancer cells, but also on healthy cells. However, recent findings suggest that naturally derived secondary metabolites from plants are well known to exhibit selective cytotoxicity toward cancer cells and minimal toxicity towards normal cells [4]. Intense research into finding out the new cancer treatment targets has led to the identification of Bcl-2 overexpression as a hallmark for cancer and Bcl-2 inhibition as a possible cancer treatment approach [5].

The Bcl-2 protein inhibition is a promising therapeutic target for developing new anticancer drugs because it is a key regulator of the intrinsic apoptosis pathway involved in various cancers [3,6]. The Bcl-2 inhibitors offer a validated pre-clinical platform for testing new therapeutic drugs and have been used to treat multiple cancers. Therefore, this protein can be a potential target receptor for developing new therapeutic agents to treat various cancers. The literature reveals that numerous Bcl-2 targeting experimental drugs (such as Obatoclax, Gossypol, Oblimersen, Epigallocatechin-3-gallate, and Navitoclax) (Supplementary Figure 1) are still now undergoing clinical studies [2]. Hence, it is of further interest to identify the new Bcl-2 inhibitors from the plant-derived compounds. The molecular docking study is widely used as a powerful tool in the pharmaceutical industry, particularly in the analysis of protein structure activity relationships [7,8,9]. It plays a vital role in predicting the molecular interactions of small ligand molecules with appropriate binding sites of the targeted protein. Moreover, molecular dynamics simulations can be used to investigate the conformational changes that occur during protein–ligand interactions. In addition to the intrinsic pathway, the extrinsic pathway is activated by tumor necrosis factor (TNF), which triggers apoptosis by binding to its death receptors. The selective TNF receptors can presently be identified as one of the novel drug targets for the treatment of cancer [10].

*Wedelia trilobata*, also known as *Sphagneticola trilobata* (L.), is a traditionally used medicinal plant renowned for its therapeutic properties in the treatment of various diseases [11]. The plant is a rich source of natural medicines that can be used as hepatoprotective, anti-inflammatory, antimicrobial, antioxidant, anti-malarial, anticancer, and antidiabetic agents [12,13,14]. In the present study, the bioactive secondary metabolites from *W. trilobata* were explored to identify their inhibitory potential against the anti-apoptotic Bcl-2 protein associated with cancer cell survival and resistance by employing the in silico computational methods like—molecular docking, molecular dynamics simulation, and ADMET analysis).

## 2. Results and Discussion

**Figure 1:**
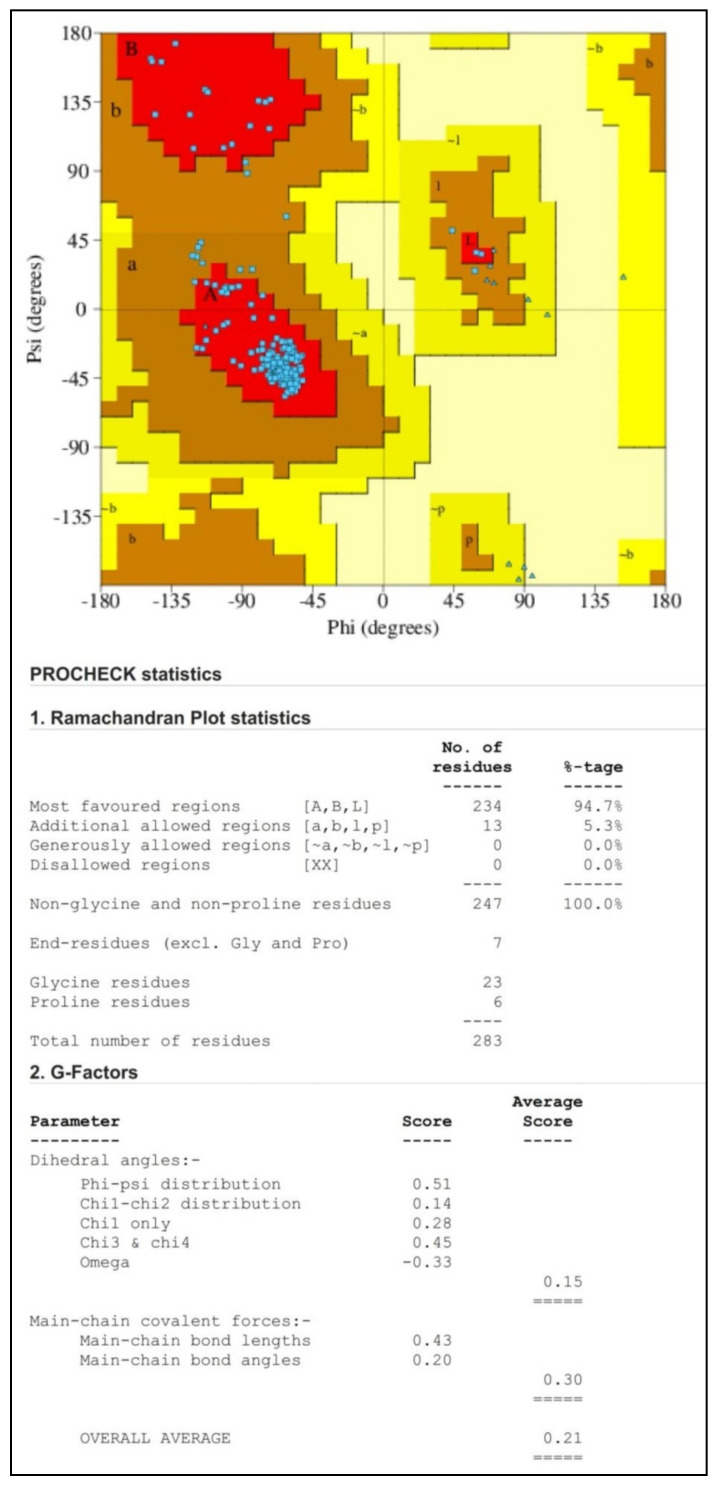
Ramachandran plot analysis of Bcl-2 protein (PDB: 2W3L) structure generated by the PROCHECK web server. The red color regions in the graph indicate protein residues in the most allowed regions; the brown color regions indicate the residues in additional allowed regions; the yellow color regions indicate the residues in generously allowed regions.

**Figure 2:**
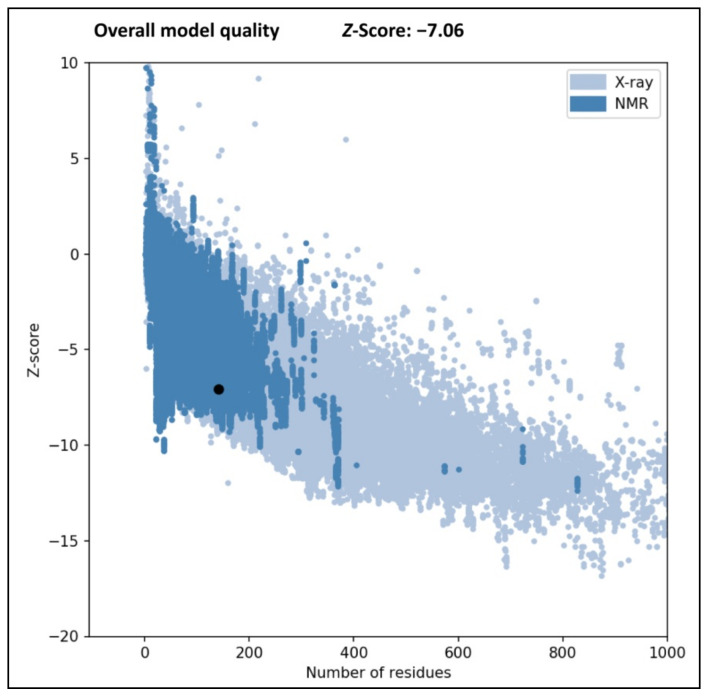
Z-score for the Bcl-2 protein (PDB: 2W3L) structure generated by ProSA web server.

The Bcl-2 is an important regulatory protein of apoptosis or programmed cell death that regulates the fundamental biological process by the formation of heterodimers with selective pro-apoptotic Bcl-2 family members [1]. Preventing the targeted anti-apoptotic Bcl-2 protein in the present study is the key to stimulating the apoptosis process in cancer cells. In the present study, in silico computational methods can be exploited to build the appropriate drug targets as well as to design novel therapeutic agents to block the apoptosis regulator Bcl-2. However, the ideal criteria for each drug in the docking techniques are that it should have the highest binding affinity for inhibiting the Bcl-2 protein. The obtained PROCHECK plot showed that most of the amino acid residues of Bcl-2 protein are found within their most favored regions (Figure 1). The Z-score generated was found within the range of its native protein, which validates that the protein model used is high quality (Figure 2). The binding sites of the structure of chimaeric Bcl2-xL (PDB: 2W3L) with phenyl tetrahydroisoquinoline amide complex is given as Supplementary Figure S2.

### 2.1. Molecular Docking Study

**Figure 3:**
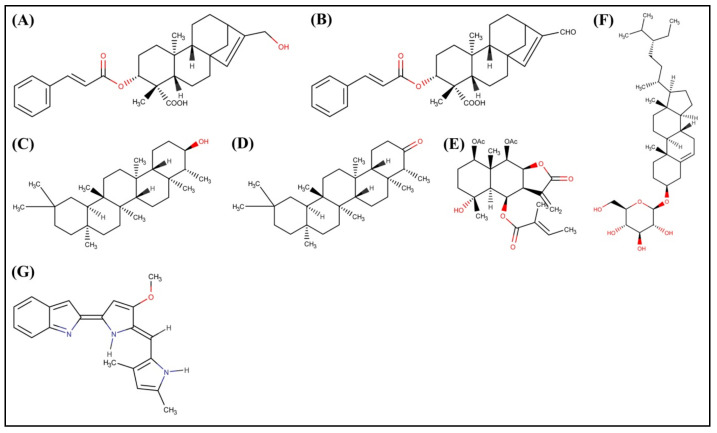
Chemical structure of the top potential phytochemicals from *W. trilobata* and standard drug. (**A**) Wedelidin A; (**B**) Wedelidin B; (**C**) Friedelinol; (**D**) Friedelin; (**E**) Trilobolide 6-O-angelate; (**F**) Daucosterol; and (**G**) Obatoclax.

**Figure 4:**
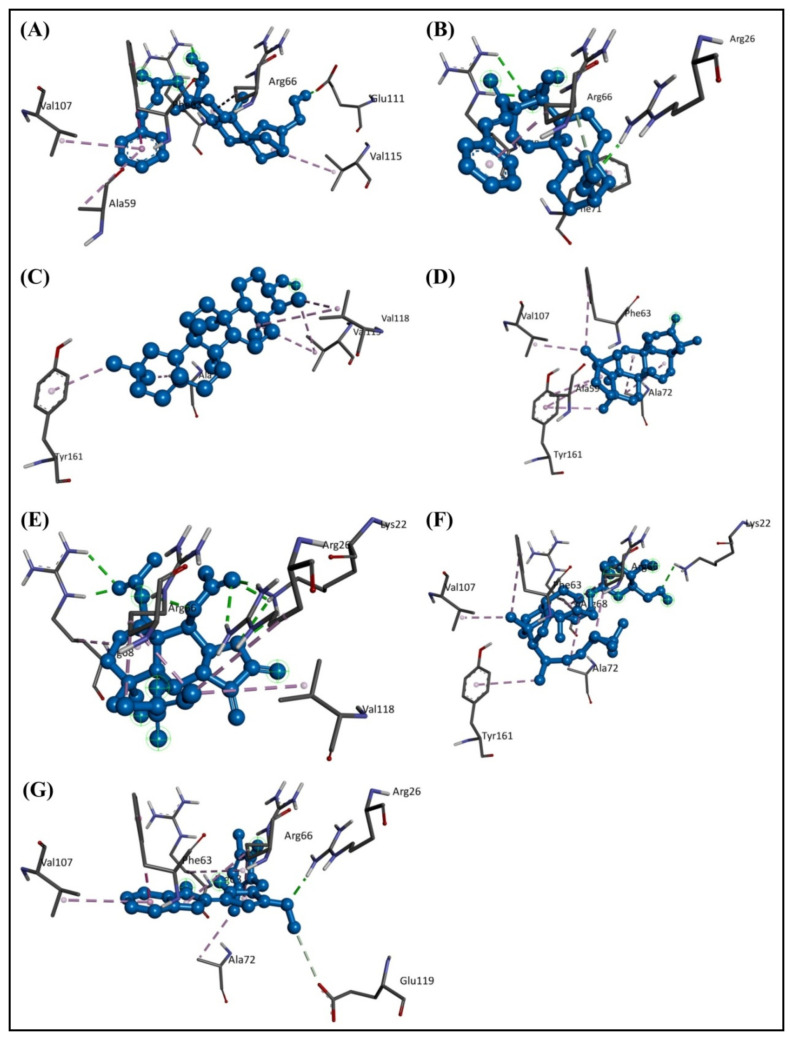
Three-dimensional illustration of the interaction of Bcl-2 protein with the top potential phytochemicals from *W. trilobata* and standard drug. (**A**) Wedelidin A; (**B**) Wedelidin B; (**C**) Friedelinol; (**D**) Friedelin; (**E**) Trilobolide 6-O-angelate; (**F**) Daucosterol; and (**G**) Obatoclax. The hydrogen bonds and hydrophobic interaction are depicted by green and purple dashed lines, respectively.

**Figure 5:**
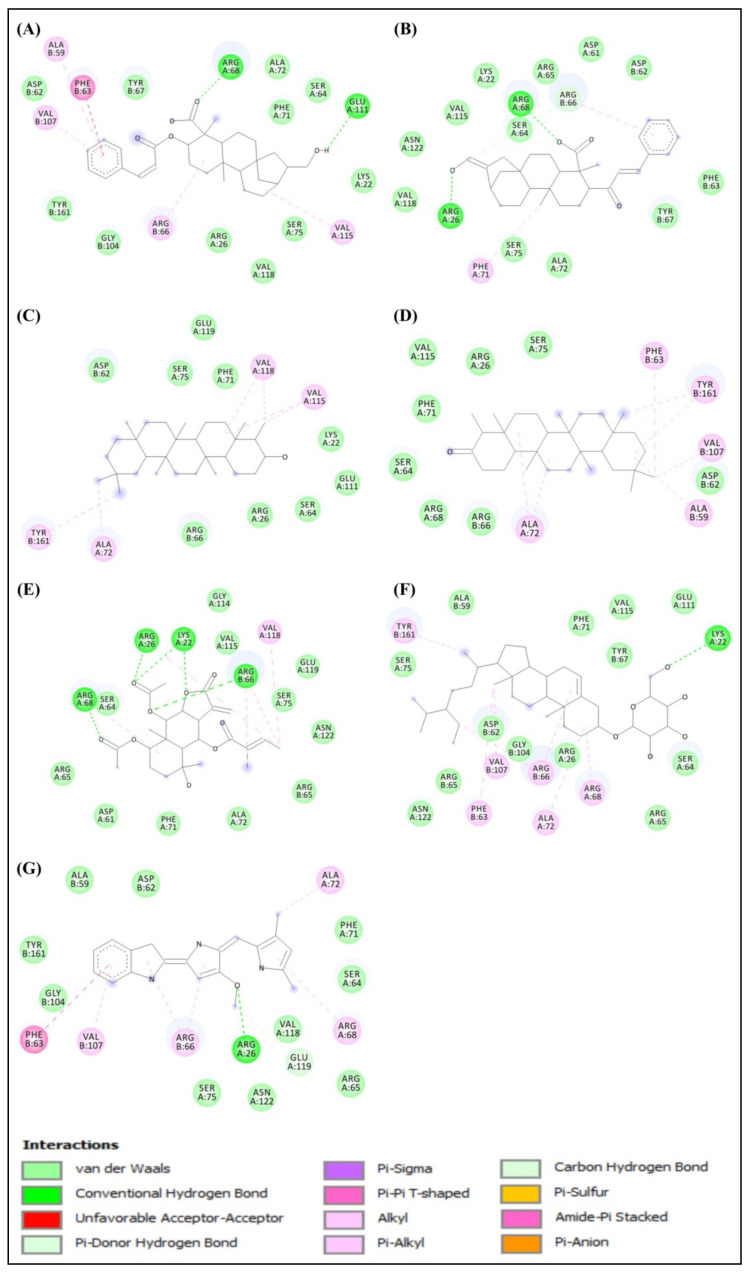
Two-dimensional illustration of the interaction of Bcl-2 protein with the top potential phytochemicals from *W. trilobata* and standard drug. (**A**) Wedelidin A; (**B**) Wedelidin B; (**C**) Friedelinol; (**D**) Friedelin; (**E**) Trilobolide 6-O-angelate; (**F**) Daucosterol; and (**G**) Obatoclax.

The docked results showed that among the bioactive secondary metabolites of *W. trilobata*, Friedelin (−10.1 kcal/mol) has the best binding affinity toward the target protein, followed by Friedelinol (−9.6 kcal/mol) in comparison with the remaining metabolites and the standard drug Obatoclax (−8.4 kcal/mol). The chemical structures of the top potential metabolites from *W. trilobata* and the standard drug are represented in Figure 3. The molecular docking analysis data of the bioactive secondary metabolites from *W. trilobata* against Bcl-2 protein are presented in Table 1. The detailed interaction (2D and 3D) between the selected docked compounds and Bcl-2 protein is shown in Figure 4 and Figure 5.

Generally, a good inhibitor has a sufficient number of hydrogen bonds and other hydrophobic interactions, which are very important for protein–ligand interactions [15]. During protein–ligand interaction, new hydrogen bonds are formed between them by breaking the existing hydrogen bonds with water molecules. The residues ALA59, PHE63, ALA72, VAL107, and TYR161 of the Bcl-2 protein were found to interact with the Friedelin of *W. trilobata* (Figure 5). Therefore, the optimal binding features of the Friedelin of *W. trilobata* and Obatoclax with the Bcl-2 protein are supported for further study and validation through molecular dynamics simulation. Adewole and Ishola [16] have reported that six compounds (Campesterol, Cycloartenol, Oleanolic acid, β-Sitosterol, Stigmasterol, and Ursolic acid) identified from 47 compounds in *Morinda lucida* Benth were found to display higher binding affinities towards Bcl-2 (PDB: 2W3L) than the reference ligand Obatoclax. In addition to hydrogen bond interaction, the hydrophobic interaction was also predominantly involved in the strong binding of potential compounds with binding pockets of Bcl-2. In addition, Mohamad Rosdi et al. [17] have reported that the pre-clinical Bcl-2 inhibitor Obatoclax was used for comparison of the bioactive compounds from *Annona muricata* with the binding energy of −7.01 kcal/mol, including interacting residues of Phe-109, Phe-101, Met-112, Val-130, Leu-134, Gly-142, Arg-143, Ala-146, Phe-150, and Val-153.

### 2.2. Structural Activity Relationship Analysis

**Figure 6:**
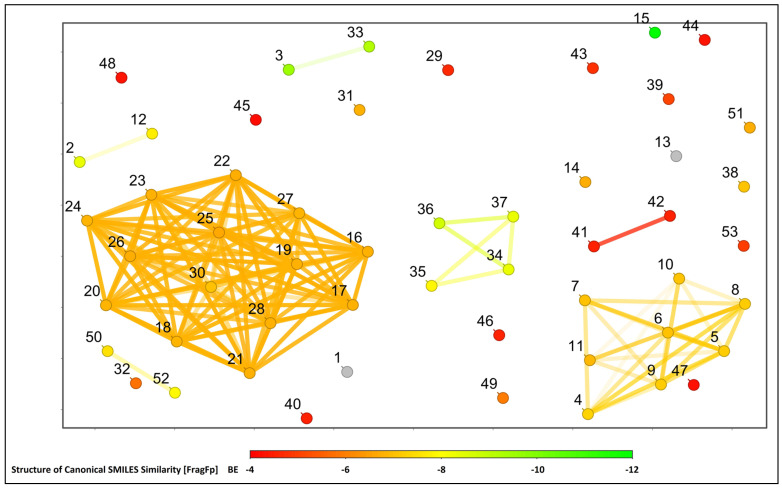
The image represents the structural activity relationship of 52 molecules and a standard drug undertaken during the study. Lines between dots represent structurally similar molecules, and the number on each dot represents the name of the molecule, as listed in Table 1.

All the selected compounds and Obatoclax were selected against the Bcl-2 protein for further structural similarity and activity relationship analysis based on the correlation of canonical SMILES structure similarity and the binding energy of selected compounds with the help of Data Warrior software. The structurally similar compounds were found to be in a group with a similar range of binding affinity values (Figure 6).

### 2.3. Molecular Dynamics Simulation Studies

It is well established that molecular dynamics simulations can be used as powerful tools for studying biological processes at the atomic level, such as protein conformational stability, flexibility, and behavior that occurs under different physiological conditions [18,19]. In the current study, the molecular dynamics simulations were executed to determine the structural stability of the docked complex of the Bcl-2 protein with Friedelin and Obatoclax within the time frame of 100 ns. The 2W3L complexed with Friedelin was selected based on its lower binding energy, while the 2W3L complexed with the standard drug Obatoclax was also considered in the present study to obtain a better comparison. Thus, both the selected complexes were subjected to the molecular dynamics simulations at 100 ns.

**Figure 7:**
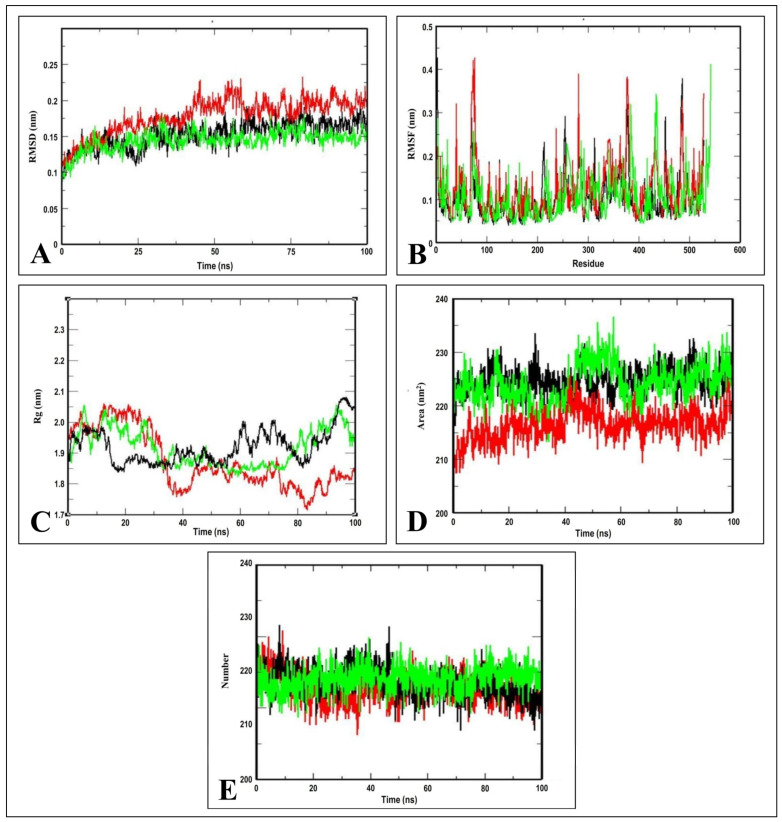
Molecular dynamics simulation of 2W3L protein alone (black), 2W3L–Friedelin complex (green), and 2W3L–Obatoclax complex (red). (**A**) RMSD; (**B**) RMSF; (**C**) Radius of gyration; (**D**) SASA; and (**E**) Hydrogen bond plots.

The root mean square deviation (RMSD), which is calculated for the atoms in the complexes’ backbones, is an important parameter for analyzing the equilibration of molecular dynamics trajectories [20]. The measurement of the RMSD backbone for two complexes offered information regarding the conformational stability. The 2W3L–Friedelin complex maintained a stable RMSD profile throughout the rest of simulation periods, and with no over-fluctuation, demonstrating the rigid conformation. However, the 2W3L–Obatoclax complex showed a similar trend of RMSD profile until 15 ns, and thereafter it increased (Figure 7A). The root mean square fluctuation (RMSF) analysis was carried out to estimate the average fluctuations of protein residues during molecular dynamics simulation [21]. The RMSF plots of the 2W3L–Friedelin complex were predicted with fluctuations only at the loop regions and terminal ends compared to the 2W3L–Obatoclax complex, which showed more fluctuations, thus indicating the stable interactions between 2W3L–Friedelin complex (Figure 7B).

The radius of gyration (Rg) considers the varied masses when calculating the root mean square distances with the rotation of the central axis. As the simulation progresses, it considers the protein folding, shape, and capability of the entire trajectory at each time step. The Rg plots of the protein alone and protein–ligand complex, also equilibrated within 1.85–2.0 nm, indicating the complex stability (Figure 7C). The region surrounding the hydrophobic core generated by the protein–ligand interaction is measured by the solvent accessible surface area (SASA). The SASA plots of protein alone and protein–ligand complex were calculated with the value of 215–235 nm^2^, indicating the consistency in the formation of the complexes (Figure 7D). The intermolecular hydrogen bonds are ubiquitous and play a vital role in protein folding and protein–ligand interactions [15]. The stability of the hydrogen bond network formed in the protein–ligand complex was calculated throughout the simulation period of 100 ns. The total number of hydrogen bonds in complexes versus time at 300 K can be seen in Figure 7E. The 2W3L–Friedelin complex exhibited the required amount of hydrogen bonds throughout the simulation, indicating that Friedelin has stable and strong hydrogen bonds with the 2W3L protein.

### 2.4. Binding Free Energy Calculation

During molecular dynamics simulations, a variety of energy metrics, including electrostatic, van der Waal’s, SASA, polar solvation, and binding energy, were used to estimate the strength of the interaction between the ligand and target protein. In this study, the van der Waal energy, electrostatic energy, SASA energy, and binding energy were mainly used to form the protein–ligand complex. As the numbers were positive, the polar solvation energy was predicted to have no effect on the formation of the protein–ligand complex. The 2W3L complexed with Friedelin, along with the standard drug Obatoclax, were considered for their binding energy calculation. The standard deviation of the protein–ligand complex was also computed. A higher standard deviation indicates that the data values are dispersed over a larger range, whereas a lower standard deviation indicates that the data values are closer to the mean (or expected value). However, there was no higher standard deviation in the Friedelin-receptor complex compared to Obatoclax, which has high standard deviation values, thereby indicating that Friedelin strongly binds to the 2W3L protein with higher binding affinity and stable interaction. The binding free energy calculation of the 2W3L target protein complexed with Friedelin and Obatoclax are represented in Table 2.

### 2.5. Prediction of ADMET Properties of Ligand Molecules

The main issue associated with the further development of novel chemical compounds into therapeutic drugs is their poor pharmacokinetics, including their physicochemical properties and ADMET properties [22]. The ADMET properties are phenomena that are directly connected to how a chemical substance behaves inside the human body. Each of the properties of ADMET will depict what happens when a chemical substance interacts with different organs in the body. The predicted ADMET properties of compounds are crucial, especially for foreign chemical compounds consumed in high doses or over extended periods. Information regarding the ADMET properties of chemical compounds is primarily required to develop novel drug compounds. Computational model-based predictions serve as virtual filters for drug-like features and help medicinal chemists to design drug candidates rationally [22,23].

The molecular polar surface area (PSA) is a valuable characteristic that is strongly related to drug absorption [24]. The PSA of Friedelin and Obatoclax were >140 Å^2^, indicating that they showed a strong polarity and were not easily absorbed by the body. Lipophilicity is the most important physicochemical characteristic of substances, connected to their solubility and human intestinal membrane permeability [25]. It was noticed that Friedelin was considered as poor lipophilicity as it displayed logP ≥5 compared to Obatoclax. The result suggested that Friedelin has poor absorption and permeation abilities. Friedelin was predicted to show moderately high Caco-2 permeability and was found to absorb more easily via the human intestine than Obatoclax. Compared to Obatoclax, Friedelin was considered to show relatively low skin permeability. The results suggested that Friedelin was not a P-glycoprotein substrate but was found to be a P-glycoprotein inhibitor when compared to Obatoclax.

The predicted results also showed that the volume of distribution at steady state (VDss) and fraction unbound (Fu) in Friedelin’s plasma was lower than Obatoclax. The Friedelin, showing log BB of 0.72, was considered to cross the blood–brain barrier easily compared to Obatoclax and could penetrate the central nervous system. Friedelin was the only substrate for CYP3A4 and was not an inhibitor of Cytochromes P450 (CYPs). The total clearance of Friedelin was found to be the lowest and not a renal OCT2 substrate. Friedelin may not be toxic in the AMES test, but proved to have the highest oral rat acute toxicity with LD_50_ of 2.64 mol/kg. Friedelin may not be hepatotoxic compared to Obatoclax, but it did not have any skin sensitizing potency. Friedelin showed the lowest *Tetrahymena pyriformis* toxicity (0.3 μg/L) and minnow toxicity (−2.384 mM) compared to Obatoclax. Therefore, the predicted results indicated that Friedelin’s physicochemical and ADMET properties supported the in silico computational studies compared to the drug Obatoclax (Supplementary Table S1).

## 3. Materials and Methods

### 3.1. Preparation of Ligands

For the present study, the ligand molecules were selected based on the three-dimensional (3D) chemical structures of 52 bioactive secondary metabolites from *W. trilobata* [26]. In addition, the anti-apoptotic known inhibitor Obatoclax was used as the standard drug for the present study. All of the selected phytochemicals’ chemical structures were retrieved in structured data format (SDF) from the PubChem compound database [27], and those that were not available in the database were drawn using Chemaxon’s chemical drawing tool (MarvinSketch version 18.30). OpenBabel version 2.3.2 was utilized to prepare the ligands, which were then exported in protein data bank (PDB) format [23]. The geometries of ligand PDB files were optimized using the PRODRG server before molecular docking.

### 3.2. Preparation of Bcl-2 Protein Structure

Based on the literature, in the present study, the crystal structure of chimeric Bcl2-xL and phenyl tetrahydroisoquinoline amide complex with the PDB ID: 2W3L [16] was selected as the main therapeutic target protein. The 3D X-Ray crystallographic structure of the target protein was retrieved in PDB format from Research Collaboratory for Structural Bioinformatics Protein Data Bank (RCSB PDB) database [28]. For further docking studies, the protein structure was processed by removing the co-crystallized ligand and water molecules bound to it and subsequent addition of hydrogens and Kollmann charges [29]. Swiss-PDB Viewer software (version 4.10) was used to perform the energy minimization of the protein structure, with empirical force fields to generate its lower energy conformation, which suggests a more stable conformation. While modeling the protein structure, the procedure essentially optimized the conformational errors in the structure’s geometry. In addition, the geometry optimization method used the steepest descent algorithm with the GROMOS96 force field [30].

### 3.3. Validation of Protein Structure

The model structure of the Bcl-2 protein was further validated in the PROCHECK online server using the Ramachandran plot, which inferred the reliability of the protein structure model developed in the SWISS MODEL server. Each amino acid included in the protein may be visualized in the plot, along with its highly favored, preferred, and disallowed phi (φ) and psi (ψ) dihedral angles in degrees [18]. The Protein Structure Analysis (ProSA) web tool was also used to check the protein structure and analyze the protein model quality. In addition, the selected protein was validated as high-quality when its Z-score fell within the range of its corresponding native protein.

### 3.4. Molecular Docking Analysis

The molecular docking experiment was executed using AutoDock Vina software integrated in the PyRx 0.8 tool, based on the Lamarckian Genetic Algorithm, to study the ligand interaction with the active site residues of Bcl-2 structure [31,32]. The cubical grid box dimension for the XYZ coordinates was fixed at 60 × 60 × 60 Å with 0.375 Å around the active binding sites of the 2W3L protein. The whole protein was surrounded within the dimensions of a grid box with docking exhaustiveness of 100 poses. The docking parameters were set to default, and the protein coordinates were saved in pdbqt file format. The docking results were represented in binding affinities expressed in kilocalories per mole (kcal/mol). The best docking pose was the conformation with the lowest binding energy. After docking, the best binding poses of each ligand molecule were visualized, and their interactions with the Bcl-2 protein structure were analyzed using BIOVIA Discovery Studio Visualizer [33]. The re-docking (self-docking) was performed with the best compounds and Bcl-2 protein to validate the accuracy of the docking methodology.

### 3.5. Structural Activity Relationship Analysis

The Data Warrior software (Version 5.5.0) was used to study the structural activity relationship analysis of the compounds used in the study, with the Bcl-2 protein based on their canonical SMILES and binding energy [34,35,36].

### 3.6. Molecular Dynamics Simulation

The 2W3L–Friedelin complex, which showed the lowest binding energy, was further subjected to the molecular dynamics simulation compared with the 2W3L–Obatoclax complex as a reference protein–ligand complex. The molecular dynamics simulations were executed with GROningen MAchine for Chemical Simulations (GROMACS) version 2018.1 using a CHARMM36 force field to confirm the conformational stability of identified ligand molecule with the Bcl-2 protein [37,38]. The ligand topology files were generated with the help of the CGenFF server [39]. Initially, the pdb2gmx module of the GROMACS was utilized to add hydrogens to the system. The protein–ligand complex was then solvated by placing the complex in a pre-equilibrated rhombic dodecahedron (RHDO) solvent box with a minimum distance of 10 Å from the box boundaries. The dodecahedral simulation box model was filled up with the TIP3P water molecules. The solvated system was then neutralized by adding an adequate number of counter ions (Na^+^ or Cl^−^) and co-ions to maintain the complex system’s electroneutrality states. The steepest descent method was employed for the energy minimization of systems to 1000 steps to remove the short contacts and atoms overlaps. The system was equilibrated for 100 ps at 300 K under constant pressure conditions in the canonical ensemble (NVT) and isothermal-isobaric (NPT) ensemble. Finally, the molecular dynamics simulations were executed at 100 ns, with an integration time of 0.002 ps under the same conditions [40].

### 3.7. Binding Free Energy Calculation

The outcome of the molecular dynamics simulation run for the target protein complexed with Friedelin and standard Obatoclax was subjected to the calculation of binding free energy using the Molecular Mechanics Poisson-Boltzmann (Generalized-Born) Surface Area (MM-PBSA) approach. The thermodynamic cycle is typically used to calculate the protein–ligand binding free energy based on the molecular dynamics simulations. The gmx_MMPBSA which uses the GROMACS 2018.1 trajectories was used to calculate the binding free energy for each protein–ligand complex [41]. The binding free energy was calculated by the gmx_MMPBSA program using three main components: molecular mechanical energy, polar solvation energy, and apolar solvation energy. The calculations were based on the molecular dynamics trajectories of the last 100 ns, which compute ∆G with dt 1000 frames. The free binding energy calculation using Equations (1) and (2) is shown below.

ΔG_Binding_ = G_Complex_ − (G_Protein_ + G_Ligand_) (1)

ΔG = ΔE MM + ΔG Solvation − TΔS = ΔE (Bonded + Non-bonded) + ΔG (Polar + Non-polar) − TΔS (2)

G_Binding_, binding free energy; G_Complex_, total free energy of protein–ligand complex; G_Protein_ and G_Ligand_, total free energies of protein and ligand in solvent, respectively; ∆G, standard free energy; ∆E MM, average molecular mechanics potential energy in vacuum; ∆G Solvation, solvation energy; ∆E, total energy of bonded as well as non-bonded interactions; ∆S, change in entropy of system upon ligand binding; and T, temperature expressed in Kelvin.

### 3.8. Prediction of ADMET Properties of Ligands

The early prediction of ADMET profiles of chemically synthesized and eco-friendly pharmaceutical drugs has revolutionized disease management methods using the most popular open access in silico tools. The pkCSM database server was used to determine the physicochemical properties and ADMET properties of target ligand molecules, which facilitated identifying promising drug candidate molecules [23,42].

## 4. Conclusions

In summary, the molecular docking study suggested that the binding orientation of Friedelin of *W. trilobata* within the binding sites of the Bcl-2 protein resulted in inhibition of enzyme activity compared to other metabolites and Obatoclax (as a standard drug). The molecular dynamics simulation studies showed that the 2W3L–Friedelin complex maintained stable RMSD and Rg profiles, along with strong hydrogen bonds throughout the simulation period of 100 ns compared to the 2W3L–Obatoclax complex. The pkCSM provided the predicted ADMET properties of Friedelin, which are relatively better than Obatoclax, wherein Friedelin has no AMES toxicity, hepatotoxicity, or skin sensitization. This study concluded that Friedelin of *W. trilobata* is one of the good inhibitory compounds of the Bcl-2 protein. Therefore, it seems that the improved binding features of Friedelin with the Bcl-2 protein supports further consideration towards in vitro and in vivo validation before clinical trials.

## Figures and Tables

**Table 1:** Molecular docking results of phytochemicals from *W. trilobata* and standard drug against Bcl-2 protein.

Sl. No.	Compound Name	Binding Energy (kcal/mol)
1.	Kaurenoic acid	−8.5
2.	Grandiflorenic acid	−7.7
3.	ent-kaura-9(11), 16-dien-19 oic acid methyl ester	−7.6
4.	3alpha-(senecioyloxy)-ent-kaur-16-en-19 oic acid	−8.6
5.	(3alpha)-3-(angeloyloxy)-ent-kaur-16-en-19 oic acid	−8.0
6.	3alpha-(angeloyloxy) 9beta-hydroxy-ent-kaur-16- en-19 oic acid	−8.1
7.	Methyl-3alpha-(angeloyloxy) 9beta-hydroxy-entkaurenoate	−8.4
8.	(3alpha)-3-(tiglinoyloxy)-ent-kaur-16-en-19 oic acid	−7.9
9.	(3alpha)-3-(cinnamoyloxy)-entkaur-16-en-19 oic acid	−8.7
10.	15alpha-(cinnamoyloxy)-ent-kaur-16-en-19 oic acid	−8.6
11.	Wedelidin A	−9.4
12.	Wedelidin B	−9.2
13.	Wedelia-seco-kaurenolide	−7.7
14.	Friedelinol	−9.6
15.	Friedelin	−10.1
16.	1beta, 4alpha-dihydroxy-6beta-isobutyryloxy-9alpha-(tigloyloxy) prostatolide	−8.8
17.	9alpha-(angeloyloxy)-1beta, 4alpha-dihydroxy-6 beta-isobutyryloxyprostatolide	−8.6
18.	1beta, 9alpha-diacetoxy-4alpha hydroxy-6beta-isobutyryloxy prostatolide	−8.5
19.	1beta, 9alpha-diacetoxy-4alpha hydroxy-6beta-methacryloxy prostatolide	−8.3
20.	Wedeliatrilolactone A	−8.4
21.	Wedeliatrilolactone B	−8.0
22.	Trilobolide 6-O-angelate	−9.1
23.	Trilobolide 6-O-methacrylate	−8.5
24.	Oxidoisotrilobolide 6-O-isobutyrate	−7.5
25.	Oxidoisotrilobolide 6-O-angelate	−7.8
26.	Oxidoisotrilobolide 6-O-methacrylate	−7.7
27.	Wedelolide A	−8.2
28.	Wedelolide B	−8.3
29.	Wedelolactone	−8.1
30.	Ivalin	−7.0
31.	Germacrene D	−6.4
32.	alpha-humulene	−6.8
33.	Caryophyllene	−6.8
34.	Stigmasterol	−8.5
35.	(7alpha)-7-hydroxystigmasterol	−8.3
36.	(3beta)-3-hydroxy stigmasta-5, 22-dien7-one	−8.3
37.	Sitosterol	−8.0
38.	Daucosterol	−9.0
39.	Squalene	−7.6
40.	3-hydroxy-6-methoxychromen-4-one	−5.8
41.	Apigenin	−7.7
42.	Diosmentin	−7.3
43.	Benzeneacetic acid 2-phenylethenyl ester	−7.1
44.	Isocinnamic acid	−5.9
45.	4-methoxy catechol	−5.3
46.	p-cymene	−6.1
47.	Protocatechualdehyde	−5.6
48.	Caffeic acid	−6.6
49.	alpha-phellandrene	−5.5
50.	alpha-pinene	−5.7
51.	D-limonene	−5.5
52.	Bicyclogermacrene	−6.6
53.	Obatoclax [Standard drug]	−8.4

**Table 2:** Binding free energy calculation of 2W3L target protein complexed with Friedelin and Obatoclax.

Categories	2W3L—Friedelin Complex		2W3L—Obatoclax Complex	
	Values (kcal/mol)	Standard Deviation (kcal/mol)	Values (kcal/mol)	Standard Deviation (kcal/mol)
Van der Waal’s energy	−138.486	±36.214	−210.986	±62.264
Electrostatic energy	−14.698	±9.246	−58.677	±17.622
Polar salvation energy	82.732	±33.218	89.918	±29.725
SASA energy	−16.140	±6.982	56.809	±18.264
Binding energy	−106.362	±36.542	−229.323	±58.825

## Data Availability

The data presented in this study are available within the manuscript.

## Supplementary Materials

The following supporting information can be downloaded at: https://www.mdpi.com/article/10.3390/molecules28041588/s1, Supplementary Table S1. ADMET properties of the selected compounds. Supplementary Figure S1. Structures of Bcl-2 targeting experimental drugs under clinical studies. (A) Gossypol, (B) Epigallocatechin-3-gallate, and (C) Navitoclax. Supplementary Figure S2. Binding sites of structure of chimaeric Bcl2-xL (PDB: 2W3L) with phenyl tetrahydroisoquinoline amide complex.

## References

[1] Belka C., Budach W. (2002). Anti-apoptotic Bcl-2 proteins: Structure, function and relevance for radiation biology. Int. J. Radiat. Biol..

[2] Alam M., Ali S., Mohammad T., Hasan G.M., Yadav D.K., Hassan M.I. (2021). B Cell lymphoma 2: A potential therapeutic target for cancer therapy. Int. J. Mol. Sci..

[3] Porter J., Payne A., de Candole B., Ford D., Hutchinson B., Trevitt G., Turner J., Edwards C., Watkins C., Whitcombe I. (2009). Tetrahydroisoquinoline amide substituted phenyl pyrazoles as selective Bcl-2 inhibitors. Bioorg. Med. Chem. Lett..

[4] Lichota A., Gwozdzinski K. (2018). Anticancer Activity of Natural Compounds from Plant and Marine Environment. Int. J. Mol. Sci..

[5] García-Aranda M., Pérez-Ruiz E., Redondo M. (2018). Bcl-2 Inhibition to Overcome Resistance to Chemo- and Immunotherapy. Int. J. Mol. Sci..

[6] Kapoor I., Bodo J., Hill B.T., His E.D., Almasan A. (2020). Targeting BCL-2 in B-cell malignancies and overcoming therapeutic resistance. Cell Death Dis..

[7] Puttaswamy H., Gowtham H.G., Ojha M.D., Yadav A., Choudhir G., Raguraman V., Kongkham B., Selvaraju K., Shareef S., Gehlot P. (2020). In silico studies evidenced the role of structurally diverse plant secondary metabolites in reducing SARS-CoV-2 pathogenesis. Sci. Rep..

[8] Murali M., Gowtham H.G., Ansari M.A., Alomary M.N., Alghamdi S., Almehmadi M., Singh S.B., Shilpa N., Aiyaz M., Kalegowda N. (2022). Repositioning Therapeutics for SARS-CoV-2: Virtual Screening of Plant-based Anti-HIV Compounds as Possible Inhibitors against COVID-19 Viral RdRp. Curr. Pharm. Des..

[9] Murali M., Gowtham H.G., Shilpa N., Krishnappa H.K.N., Ledesma A.E., Jain A.S., Shati A.A., Alfaifi M.Y., Elbehairi S.E.I., Achar R.R. (2022). Exploration of Anti-HIV Phytocompounds against SARS-CoV-2 Main Protease: Structure-Based Screening, Molecular Simulation, ADME Analysis and Conceptual DFT Studies. Molecules.

[10] Fischer R., Kontermann R.E., Pfizenmaier K. (2020). Selective Targeting of TNF Receptors as a Novel Therapeutic Approach. Front. Cell Dev. Biol..

[11] Borghi S.M., Mizokami S.S., Carvalho T.T., Rasquel-Oliveira F.S., Ferraz C.R., Fattori V., Hayashida T.H., Peron J.P.S., Camilios-Neto D., Ambrosio S.R. (2021). The diterpene from *Sphagneticola trilobata* (L.) Pruski, kaurenoic acid, reduces lipopolysaccharide-induced peritonitis and pain in mice. J. Ethnopharmacol..

[12] Mizokami S.S., Arakawa N.S., Ambrosio S.R., Zarpelon A.C., Casagrande R., Cunha T.M., Ferreira S.H., Cunha F.Q., Verri W.A. (2012). Kaurenoic acid from *Sphagneticola trilobata* inhibits inflammatory pain: Effect on cytokine production and activation of the NO–cyclic GMP–protein kinase G–ATP-sensitive potassium channel signaling pathway. J. Nat. Prod..

[13] Buddhakala N., Talubmook C. (2020). Toxicity and antidiabetic activity of ethanolic extract of *Sphagneticola trilobata* (L.) *Pruski* flower in rats. J. Ethnopharmacol..

[14] Mardina V., Sufriadi E. (2020). Flower of *Sphagneticola trilobata* (L.) J.F *Pruski* from Aceh, Indonesia: Antioxidant and cytotoxic activity on HeLa cells. IOP Conf. Ser.: Mater. Sci. Eng..

[15] Coimbra J.T.S., Feghali R., Ribeiro R.P., Ramos M.J., Fernandes P.A. (2021). The Importance of Intramolecular Hydrogen Bonds on the Translocation of the Small Drug Piracetam through a Lipid Bilayer. RSC Adv..

[16] Adewole K.E., Ishola A.A. (2019). Phytosterols and triterpenes from *Morinda lucida* Benth (*Rubiaceae*) as potential inhibitors of anti-apoptotic BCL-XL, BCL-2, and MCL-1: An *in-silico* study. J. Recept. Signal Transduct..

[17] Mohamad Rosdi M.N., Mohd Arif S., Abu Bakar M.H., Razali S.A., Zulkifli R.M., Ya’akob H. (2018). Molecular docking studies of bioactive compounds from *Annona muricata* Linn as potential inhibitors for Bcl-2, Bcl-w and Mcl-1 antiapoptotic proteins. Apoptosis.

[18] Prasad S.K., Pradeep S., Shimavallu C., Kollur S.P., Syed A., Marraiki N., Egbuna C., Gaman M.-A., Kosakowska O., Cho W.C. (2021). Evaluation of *Annona muricata* Acetogenins as Potential Anti-SARS-CoV-2 Agents Through Computational Approaches. Front. Chem..

[19] Salo-Ahen O.M.H., Alanko I., Bhadane R., Bonvin A.M.J.J., Honorato R.V., Hossain S., Juffer A.H., Kabedev A., Lahtela-Kakkonen M., Larsen A.S. (2021). Molecular Dynamics Simulations in Drug Discovery and Pharmaceutical Development. Processes.

[20] Sargsyan K., Grauffel C., Lim C. (2017). How Molecular Size Impacts RMSD Applications in Molecular Dynamics Simulations. J. Chem. Theory Comput..

[21] Boroujeni M.B., Dastjerdeh M.S., Shokrgozar M.A., Rahimi H., Omidinia E. (2021). Computational driven molecular dynamics simulation of keratinocyte growth factor behavior at different pH conditions. Inform. Med. Unlocked.

[22] Sharma A., Magotra A., Dogra A., Rath S.K., Rayees S., Wazir P., Sharma S., Sangwan P.L., Singh S., Singh G. (2017). Pharmacokinetics, pharmacodynamics and safety profiling of IS01957, a preclinical candidate possessing dual activity against inflammation and nociception. Regul. Toxicol. Pharmacol..

[23] Pires D.E.V., Blundell T.L., Ascher D.B. (2015). pkCSM: Predicting small-molecule pharmacokinetic and toxicity properties using graph-based signatures. J. Med. Chem..

[24] Schaftenaar G., de Vlieg J. (2012). Quantum mechanical polar surface area. J. Comput. Aided Mol. Des..

[25] Arnott J.A., Planey S.L. (2012). The influence of lipophilicity in drug discovery and design. Expert. Opin. Drug Discov..

[26] Balekar N., Nakpheng T., Srichana T. (2014). *Wedelia trilobata* L.: A phytochemical and pharmacological review. Chiang Mai J. Sci..

[27] Kim S., Chen J., Cheng T., Gindulyte A., He J., He S., Li Q., Shoemaker B.A., Thiessen P.A., Yu B. (2019). PubChem in 2021: New data content and improved web interfaces. Nucleic Acids Res..

[28] Burley S.K., Bhikadiya C., Bi C., Bittrich S., Chen L., Crichlow G.V., Christie C.H., Dalenberg K., Costanzo L.D., Duarte J.M. (2021). RCSB Protein Data Bank: Powerful new tools for exploring 3D structures of biological macromolecules for basic and applied research and education in fundamental biology, biomedicine, biotechnology, bioengineering and energy sciences. Nucleic Acids Res..

[29] Pradeep S., Jain A.S., Dharmashekara C., Prasad S.K., Akshatha N., Pruthvish R., Amachawadi R.G., Srinivasa C., Syed A., Elgorban A.M. (2021). Synthesis, Computational Pharmacokinetics Report, Conceptual DFT-Based Calculations and Anti-Acetylcholinesterase Activity of Hydroxyapatite Nanoparticles Derived From *Acorus Calamus* Plant Extract. Front. Chem..

[30] Schwede T., Kopp J., Guex N., Peitsch M.C. (2003). SWISS-MODEL: An automated protein homology-modeling server. Nucleic Acids Res..

[31] Uppar V., Chandrashekharappa S., Shivamallu C., Sushma P., Kollur S.P., Ortega-Castro J., Frau J., Flores-Holguín N., Basarikatti A.I., Chougala M. (2021). Investigation of Antifungal Properties of Synthetic Dimethyl-4-Bromo-1-(Substituted Benzoyl) Pyrrolo [1,2-a] Quinoline-2,3-Dicarboxylates Analogues: Molecular Docking Studies and Conceptual DFT-Based Chemical Reactivity Descriptors and Pharmacokinetics Evaluation. Molecules.

[32] Gowtham H.G., Murali M., Singh S.B., Shivamallu C., Pradeep S., Shivakumar C.S., Anandan S., Thampy A., Achar R.R., Silina E. (2022). Phytoconstituents of *Withania somnifera* unveiled Ashwagandhanolide as a potential drug targeting breast cancer: Investigations through computational, molecular docking and conceptual DFT studies. PLoS ONE.

[33] Kumar V., Ramu R., Prithvi S., Shirahatti V.B., Kumari C., Sushma P., Mandal S.P., Patil S.M. (2021). α-Glucosidase, α-Amylase Inhibition, Kinetics and Docking Studies of Novel (2-Chloro-6-(trifluoromethyl)benzyloxy)arylidene) Based Rhodanine and Rhodanine Acetic Acid Derivatives. Chemistry Select..

[34] Modak R., Basha J., Bharathy N., Maity K., Mizar P., Bhat A.V., Vasudevan M., Rao V.K., Kok W.K., Natesh N. (2013). Probing p300/CBP associated factor (PCAF)-dependent pathways with a small molecule inhibitor. ACS Chem. Biol..

[35] Krishna S., Kumar S.B., Murthy T.P.K., Murahari M. (2021). Structure-based design approach of potential BCL-2 inhibitors for cancer chemotherapy. Comput. Biol. Med..

[36] Pal P., Thummuri D., Lv D., Liu X., Zhang P., Hu W., Poddar S.K., Hua N., Khan S., Yuan Y. (2021). Discovery of a Novel BCL-XL PROTAC Degrader with Enhanced BCL-2 Inhibition. J. Med. Chem..

[37] Prasad A., Shruthi G., Sushma P., Jain A.S., Chandan D., Prasad M.N.N., Kollur S.P., Srinivasa C., Shivamallu C. (2020). *Helicobacter pylori* Infection: A Bioinformatic Approach. Int. J. Pharm. Sci. Res..

[38] Dharmashekara C., Pradeep S., Prasad S.K., Jain A.S., Syed A., Prasad K.S., Patil S.S., Beelagi M.S., Srinivasa C., Shivamallu C. (2021). Virtual screening of potential phyto-candidates as therapeutic leads against SARS-CoV-2 infection. Environ. Chall..

[39] Pradeep S., Patil S.M., Dharmashekara C., Jain A., Ramu R., Shirahatti P.S., Mandal S.P., Reddy P., Srinivasa C., Patil S.S. (2022). Molecular insights into the in silico discovery of corilagin from *Terminalia chebula* as a potential dual inhibitor of SARS-CoV-2 structural proteins. J. Biomol. Struct. Dyn..

[40] Chadha N., Tiwari A.K., Kumar V., Milton M.D., Mishra A.K. (2015). In Silico Thermodynamics Stability Change Analysis Involved in BH4 Responsive Mutations in Phenylalanine hydroxylase: QM/MM and MD Simulations Analysis. J. Biomol. Struct. Dyn..

[41] Valdés-Tresanco M.S., Valdés-Tresanco M.E., Valiente P.A., Moreno E. (2021). gmx_MMPBSA: A new tool to perform end-state free energy calculations with GROMACS. J. Chem. Theory Comput..

[42] Anandan S., Gowtham H.G., Shivakumara C.S., Thampy A., Brijesh Singh S., Murali M., Shivamallu C., Pradeep S., Shilpa N., Shati A.A. (2022). Integrated approach for studying bioactive compounds from *Cladosporium* spp. against estrogen receptor alpha as breast cancer drug target. Sci. Rep..

